# SARS-CoV-2 seroprevalence among people living with HIV in the German HIV-1 Seroconverter Cohort, 2020–2022

**DOI:** 10.1186/s12879-024-10119-3

**Published:** 2024-11-01

**Authors:** Oliver Hohn, Karolin Meixenberger, Alexander Volkwein, Kyra Körner, Suheda Icli, Uwe Koppe, Martin Hower, Viviane Bremer, Barbara Gunsenheimer-Bartmeyer, Norbert Bannert, the German HIV-1 Seroconverter Study Group, Martin Hower, Heribert Knechten, Petra Panstruga, Helmut Schühlen, Caroline Isner, Hans Wesselmann, Dirk Schürmann, Ulrich Bohr, Heiko Jessen, Arne B. Jessen, Stephan Grunwald, Jan Gumprecht, Beate Weninger, Heribert Hillenbrand, Heiko Karcher, Michael Rausch, Thomas Reineke, Roland Grimm, Sarah Schoor, Michael Rittweger, Dietmar Schranz, Tobias Glaunsinger, Christiane Cordes, Hubert Schulbin, Sascha Brand, Andreas Berger, Sinah Lindemann, Ivanka Krznaric, Gordon Weinberg, Manuel Bruhy, Anja Potthoff, Adriane Skaletz-Rorowski, Thomas Grünewald, Julia Neumann, Petra Spornraft-Ragaller, Andreas Jenke, Stefan Pursche, Bjoern Jensen, Falk Hüttig, Stefan Esser, Pia Schenk-Westkamp, Annette Haberl, Christoph Stephan, Susanne Usadel, Matthias Müller, Janina Trauth, Peter Buggisch, Dorothea Wiemer, Lavinia Biemann, Ansgar Rieke, Stephan Schneeweiß, Stefan Scholten, Ulrike Haars, Jeannine Weidemann, Ines Ruck, Matthias P. Ebert, Roger Vogelmann, Johannes Bogner, Barbara Sonntag, Birgit Mück, Ramona Pauli, Christoph D. Spinner, Jochen Schneider, Nils Postel, Niels Schübel, Christiane Berning, Clemens Roll, Simone Marquardt, Steve Rößler, Cengiz Güler

**Affiliations:** 1 Department of Infectious Diseases, Unit 18 “Sexually Transmitted Bacterial Pathogens (STI) and HIV”, Robert Koch Institute, Nordufer 20, 13353 Berlin, Germany; 2 Department of Infectious Disease Epidemiology, Unit 34 “HIV/AIDS, STI and Blood-Borne Infections”, Robert Koch Institute, Seestrasse 10, Berlin, 13353 Germany; 3 Klinikum Dortmund GmbH / Klinik der Universität Witten / Herdecke, Dortmund, Germany

**Keywords:** HIV, SARS-CoV-2, Seroprevalence, Vaccination, Cohort

## Abstract

**Objectives:**

People living with HIV (PLWH) are a risk group for severe symptoms and higher mortality during COVID-19. We analyzed the dynamic rise of SARS-CoV-2 seroprevalence induced by coinfections and vaccinations in PLWH in the first three years of the pandemic in Germany and compared it with corresponding data available for the general population.

**Methods:**

Each month on average 93 blood samples from the German HIV-1 Seroconverter Cohort, a prospective longitudinal multicenter study that includes PLWH whose date of seroconversion is well defined, were received. The samples from 1569 PLWH were tested for the presence of anti-S1 and if positive, also for anti-N antibodies.

**Results:**

In 2020 the number of anti-S1 positive cases/month was between 0.0 and 6.9% (average 1.6%). Since then the anti-S1 prevalence increased reaching already 35% (33/94) in May 2021. At that time 3.2% of the cases were also anti-N positive. In 2022 the average anti-S1 seroprevalence reached 97.5%. In the vaccination era a positive anti-N response was associated with a younger age and females were overrepresented among anti-S1/anti-N negative samples (assuming no vaccination or infection).

**Conclusions:**

The average 1.6% anti-S1 seroprevalence in the cohort in 2020 was comparable to that in the general population (1.3%). The increase in anti-S1 seroprevalence in the first half of 2021 occurred slightly earlier. This increase was likely caused by the prioritization of PLWH at the early stage of the vaccination campaign and by infections during the third wave of the pandemic.

**Supplementary Information:**

The online version contains supplementary material available at 10.1186/s12879-024-10119-3.

## Introduction

In December 2019 first cases of pneumonia caused by an emerging new human betacoronavirus were reported from Wuhan, China [1, 2]. The virus that was named “Severe Acute Respiratory Syndrome Coronavirus 2 (SARS-CoV-2)” spread rapidly, leading all over the world to the Coronavirus Disease-19 (COVID-19) with a substantial fatality rate [3]. The first case in Germany was reported on January 27th 2020 [4]. The outbreak was declared a pandemic in March 2020 by the WHO [5]. A PCR-based diagnostic for SARS-CoV-2 was rapidly developed and became available for testing of nasal and throat swabs. Governments and public health officials were alarmed by initial cases in their countries and rapidly established strict regulations for containment and prevention including physical distancing, wearing of masks, shut downs in the retail sector, closings of daycare facilities as well as quarantine measures for those with a positive diagnosis and individuals that had close contacts with them.

Early in 2020 first clinical trials of SARS-CoV-2 vaccines were launched and at the end of the same year an emergency use of two mRNA vaccines was authorized by the Food and Drug Administration in the US and subsequently in Europe. The national vaccination campaign in Germany began on December 27th 2020, first prioritizing adults of age 80 and older, residents of nursing homes and certain health care workers [6]. People living with HIV (PLWH) received initially the priority level 4 (of 6 levels in total), into which also individuals at the age of 65–69 were grouped by the Standing Committee on Vaccination (STIKO) [6], and subsequently an “increased priority level” by an “Ordinance on the entitlement to vaccination” by the German ministry of health [7]. The ranking was based on evidence for an increased risk of severe or fatal COVID-19 disease, a particular professional risk or close contacts with vulnerable groups of people [6].

HIV replication and high viral loads can cause dysfunctions of immunological tissues and immunosuppression that is largely, but not fully, reversible following an effective antiretroviral therapy (ART) [8]. Despite ART, the virus is not completely eradicated and residual antigen expression induces pro-inflammatory cytokines [9]. The chronic status of immune activation and inflammation is likely responsible for tissue damage and a variety of non-AIDS related co-morbidities. The persistent immune activations impair the function of the thymus and other lymphoid tissues as well as immune cells resulting in clonal exhaustion of T-cells and immunosenescence, especially in the elderly [10]. The presumed higher vulnerability by SARS-CoV-2 and prioritization of PLWH in the vaccination campaign were supported by reports indicating a lower capacity of PLWH to respond to some pathogens and to vaccines [11, 12].

An HIV infection can have also negative effects on a coinfection. PLWH experience more severe symptoms and a faster disease progression of an HCV hepatitis or tuberculosis infection [13]. Moreover, long term ART itself increases the risk for metabolic disorders such as cardiovascular problems and hypertension that were identified as critical factors for COVID-19 severity already at the beginning of the pandemic [14].

Early in the SARS-CoV-2 pandemic reports and meta analyses of more severe symptoms and higher mortality rates in PLWH appeared justifying the higher initial vaccine prioritization [15, 16]. Moreover, in some studies lower titers of antibodies against SARS-CoV-2 were measured in HIV patients compared to HIV-negative controls after infection or vaccination [17, 18]. Apart from those reports, studies with conflicting results regarding the relative susceptibility and severity of SARS-CoV-2 infection in PLWH were published, as reviewed by del Amo and colleagues [19]. The confounders that were or were not considered and divergences in the investigated populations are discussed as a potential source of contradictory results, but data on this are rare [19–22]. There are still many open questions surrounding the reasons and the clinical parameters associated with the higher severity of COVID-19 in PLWH [23]. However, worse COVID-19 outcomes seem to correlate with the HIV viral load and low CD4 T-cell counts [21, 24].

There is also scarce and inconsistent data on differences between the dynamic of the pandemic and vaccination rates in PLWH compared to the general population. In some populations a higher SARS-CoV-2 incidence in PLWH was noticed in unadjusted analyses that disappeared after adjustment for confounders [25].

In this article we obtain data and analyze the dynamic rise of SARS-CoV-2 seroprevalence induced by coinfections and vaccinations in PLWH since the onset of the pandemic in the German HIV-1 Seroconverter Cohort. The cohort is well characterized and includes PLWH with known date of HIV infection [26]. Seroprevalence is a good estimate of the proportion of a cohort or population that had contact to the virus or received a vaccine. We compare the findings from the cohort with corresponding data available for the general German adult population.

## Material and methods

### Inclusion criteria and sampling procedure

All samples analyzed in the study that was named “HIVCOV” were obtained from participants of the German HIV-1 Seroconverter Cohort, a nationwide, multicenter, long term observational study of PLWH with known or reliably estimated date of HIV-1 infection. It was established in 1997. Participation is voluntary and inclusion criteria are (i) an acute seroconversion confirmed by laboratory diagnostics or a documented seroconversion with a shorter than 3 years interval between the last negative and the first confirmed positive HIV-1 antibody test (for details of these criteria see [26]), (ii) an age of 18 years or older, and (iii) a written consent for participation. The HIVCOV samples (EDTA blood) were provided by cohort members attending one of 47 study centers located across Germany. The blood sample was taken during a regular venipuncture recommended on a three-monthly basis for patient monitoring. One sample of a participant is submitted per calendar year to the Robert Koch Institute (RKI). The study center decides from which quarter of the year a sample is provided. In 2022 up to four samples per year (one each quarter) were received. Of these multiple samples from the same participant only the sample that matched best to one-year from the sample in 2021 was analyzed in the seroprevalence study. If no sample in 2021 was obtained, we have included the first sample received in 2022. Thus, in many cases three samples from a participant (one from each year of the analyzed period) were included in the study.

### Sample preparation

Samples were usually shipped overnight to the RKI and processed within 24 h of receipt. Plasma was isolated from EDTA-blood by centrifugation at 2000*g* for 10 min and stored in a freezer equal to minus 80 °C.

### Serologic detection of SARS-CoV-2-specific immune responses

For the detection of IgG antibodies against the SARS-CoV-2 nucleoprotein (N-protein) the “Anti-SARS-CoV-2-NCP ELISA (IgG)” from Euroimmun (Euroimmun AG, Lübeck, Germany) was used. The ELISA was performed on all anti-S1 (subunit of the viral spike protein) positive samples using the protocol recommended by the supplier. IgG responses specific for the S1-protein of SARS-CoV-2 were detected by using the “Anti-SARS-CoV-2 ELISA (IgG)” from Euroimmun. The assay was performed in accordance with the protocol of the supplier. For defining seropositivity, the ratio cut point provided by the manufacturer was used. All borderline samples were repeated and if not tested negative, counted as seropositive.

### Statistical data analysis and comparison with data from national and regional seroprevalence studies

Median and Interquartile ranges (IQR) were determined and analyzed for continuous variables and n (%) for categorical variables. Age was calculated by subtracting the year of birth from the year of the sample. *P*-values were determined by using a two-sided Student’s t-test (median age) or the Chi^2^-test within the Excel software (gender, transmission groups). Differences with *p* < 0.05 were regarded as statistically significant.

To compare the SARS-CoV-2 seroprevalence of PLWH in the HIVCOV study with the seroprevalence of the adult general population in Germany, a previously published set of results from national and local studies performed between March 2020 and March 2022 was utilized. This publication contains all data used for the comparison and is available on the RKI web page [27]. A trend line for the HIVCOV results was obtained by low-level smoothing the HIVCOV dataset (2 neighbors, 2nd order) [28] using the Graph Pad Prism software.

### Ethics

The German HIV-1 Seroconverter Cohort received ethical approvals from the ethics committee of the Charité (EA2/105/05 in 2005 and EA2/024/21 in 2021). Informed consent to participate was obtained from all of the participants in the study.

## Results

### Study population and sampling

The HIVCOV seroprevalence study for SARS-CoV-2 in PLWH was performed with samples of the German HIV-1 Seroconverter Cohort. In total 3345 samples from 1569 patients were received and examined (Table 1). Most specimens obtained in 2021 and 2022 were follow up samples from individuals already sampled in one or both of the previous years. In the second year (2021) 217/1114 (19.5%) of the samples came from new participants and in the third year of the study 85/964 (8.8%) of the samples were from individuals that have not yet contributed a sample (see Additional file 1).

**Table 1:** Patient samples

Variables	Period of time			
	**2020**	**2021**	**2022**	**2020–2022**
**Patient samples, n**	1267	1114	964	3345
**Follow up samples, n (%)**	0 (0.0)	897 (80.5)	879 (91.2)	1776 (53.1)

The median age of the participants at the time they contributed the first sample was 44.1 years (IQR 35–52) and the median years living with HIV was 9.4 [IQR 5–13]. Over 90% were on ART. The majority were male (1481/1569; 94.4%). The most common transmission groups were men who have sex with men (MSM) (1396/1569; 87.3%) followed by heterosexual transmission (HET) (125/1569; 8%) (Table 2). Least common were persons who inject drugs (PWID). Because of the high number of follow up samples, all of the mentioned proportions were largely stable over the analysis period (see Additional file 2).

**Table 2:** Study population (*n* = 1569)

Characteristic	Median	IQR
**Median age**^**a**^	44.1	35–52
**Years since HIV diagnosis**^**a**^	9.4	5–13
**Characteristic**	**n**	**%**
**On HIV therapy**^**a**^	1423	90.7^b^
**Gender**		
Male	1481	94.4
Female	73	4.7
Other or unknown	15	1.0
**Transmission group**		
MSM	1369	87.3
PWID	12	0.8
HET	125	8.0
Other or unknown	63	4.0

*Abbreviations: IQR* interquartile range, *MSM* men who have sex with men, *PWID* persons who inject drugs, *HET* heterosexual transmission

^a^At the time the first sample for the prevalence study was received

^b^Samples from new cohort inclusions can arrive before therapy has been started

### Seroprevalence of anti-SARS-CoV-2 antibodies

**Fig. 1:**
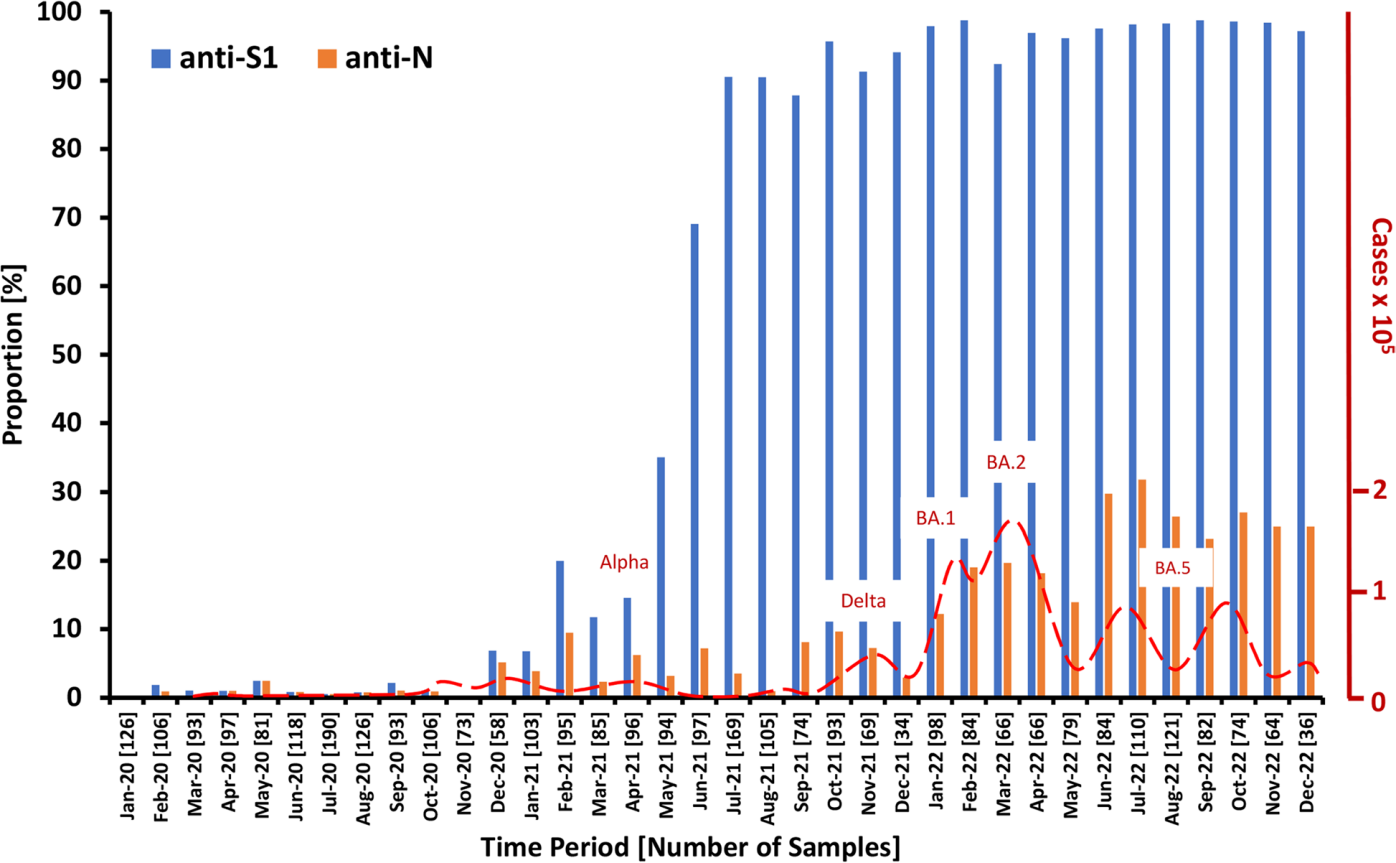
Proportion of anti-S1 and anti-N positive cases. The number of analyzed samples is given in brackets. The incidence rate in the general population in Germany is shown on the axis at the right-hand site. The incidence data are from the incidence survey of the Robert Koch Institute [29]. The dominating variant of concern in prominent infection waves is shown above the peak of a wave [30]

The 3345 samples received were analyzed on a monthly basis taking the date of arrival in the lab as time point for the analysis which was usually 1–3 days following venipuncture. In the 36-month period, the average number of samples was 93 per month but the numbers varied from 190 in July 2020 to 38 in December 2022. The first samples tested positive for anti-S1 arrived in February 2020 (Fig. 1). Up to December 2020 the proportions of samples positive for S1 antibodies were very low with a 2.5%-peak in May 2020, at the end of the first infection wave. Up to the end of 2020 all anti-S1 positive cases can be attributed to SARS-CoV-2 infections because the vaccination start in Germany was December 27th 2020. In December 2020 the proportion of anti-S1 and anti-N positive cases increased to 6.9 and 5.2, respectively, and anti-S1 positive cases increased further in the next six months. From February 2021 onwards, the difference in the proportion of anti-S1 and anti-N positive cases started to rise. The highest dynamic of the anti-S1 rise is evident in April to July. Already in June 2021 about 70% of the participant´s samples had detectable anti-S1 antibodies. Seroprevalence reached over 90% one month later followed by a slow gradual increase with a peak of 98.8% in February and September 2022. The anti-S1-seroprevalence in 2022 stayed at a high level with an average of 97.5%.

Samples tested positive for anti-S1 were also tested for anti-N responses. In 2020, 12/16 (75%) of the anti-S1-positive cases were also positive for anti-N. In 2021 and 2022 periods of increasing and decreasing proportions are discernible (Fig. 1, orange bars). The waves lag about two months behind the general incidence waves in the population that were caused by the appearance of new variants of concern (VoC). The highest anti-N seroprevalence was measured in July 2022 with 31.8%.

**Fig. 2:**
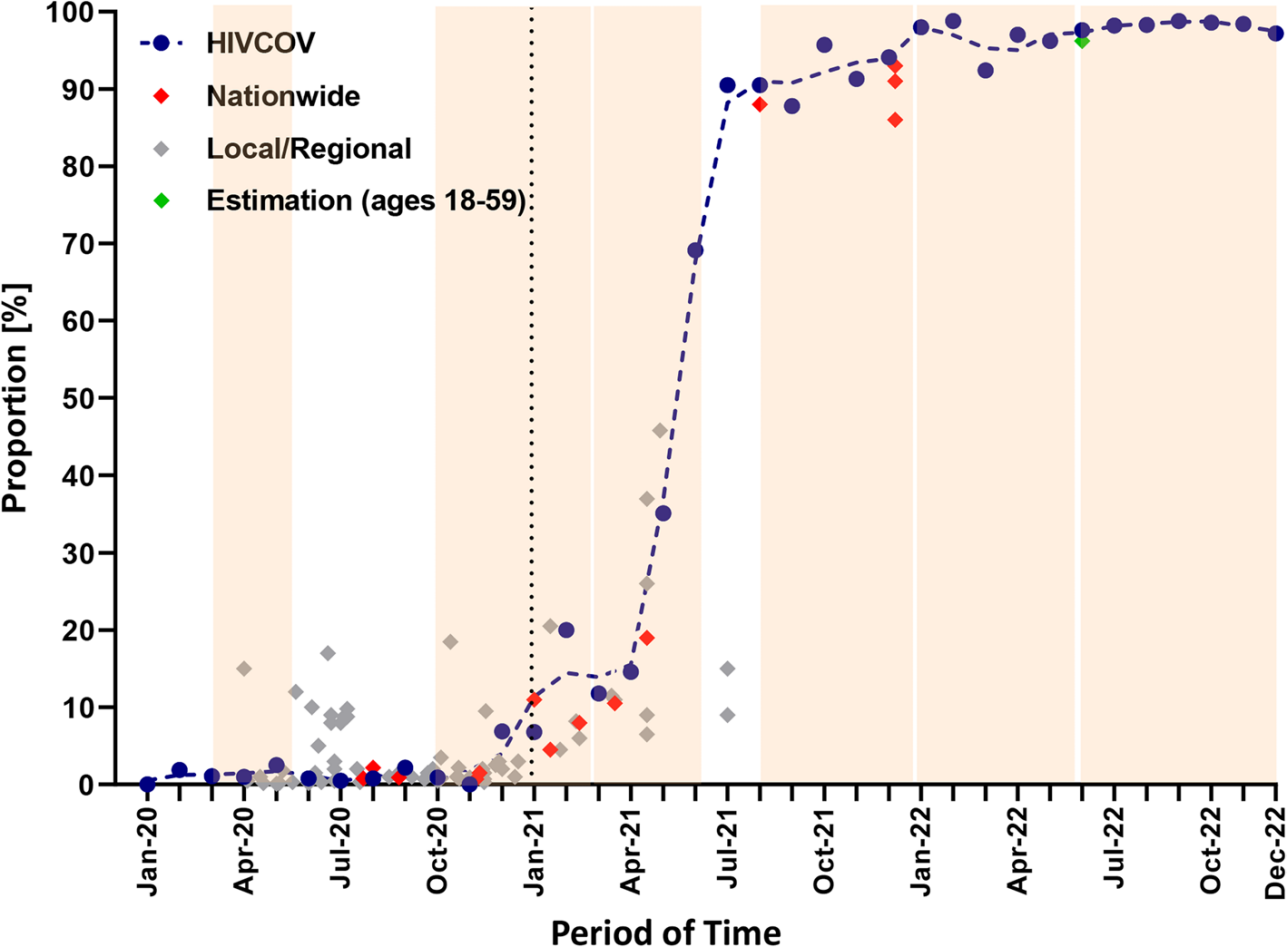
Comparison of anti-S1 seroprevalence in the HIVCOV study with data from studies of the adult general population in Germany. Data of the nationwide and local studies were previously assembled and published [27]. The dashed line indicates the trend. The dotted vertical line marks the start of the vaccination campaign. The six infection waves [30] are depicted as light brown background. The green diamond marks the estimated proportion of the German population with a previous contact to the virus or vaccine by end of May 2022, as published by Maier and colleagues [32]

A number of SARS-CoV-2 anti-S1 seroprevalence studies in the general adult population were done in Germany, mostly in the pre-vaccine era [31]. Hot-spot investigations were conducted in 2020 and early in 2021. The seroprevalence data obtained in HIVCOV and data reported in local and in nationwide studies as well as the result of a modelling approach are presented in Fig. 2.

Next, we thought to identify characteristics in the cohort that are associated with a higher risk for infection in the pre-vaccination and vaccination period of the study. For the analysis we have assigned the start of the vaccination period to February 2021 when vaccinations began to increase anti-S1 seroprevalence (see Fig. 1). For this we compared age, gender and transmission groups of anti-N seropositive cases with seronegative cases and found an about three years younger median age among anti-N positive cases during the vaccine era. (Table 3). We thought also to identify characteristics associated with SARS-CoV-2 vaccination and analyzed anti-N negative cases with or without an anti-S1 response. Anti-N-/anti-S1+ cases were assumed as vaccinated and anti-N-/anti-S1- as non-vaccinated. We found a significant overrepresentation of females in comparison with non-females (males, other genders and unknown gender) in the non-vaccinated group than in the vaccinated group (Table 3). 27.4% of the female PLWH of the HIVCOV study reported a presumed HIV-1 infection outside of Germany while this proportion is 15.5% for non-female participants.

**Table 3:** Comparison regarding serostatus and presumed vaccination

Variables	Pre vaccine era 01/20–01/21 *n* = 1294			Vaccine era 02/21–12/22 *n* = 1311					
	**N + **	**N-**^**a**^	***p* value**	**N + **	**N-**^**a**^	***p* value**	**S1-/N-**^**a**^	**S1 + /N-**	***p* value**
**Number of samples n (%)**	12 (0.93)	1282 (99.1)		123 (9.4)	1188 (90.6)		384 (29.3)	804 (61.3)	
**Median age in years** **(IQR)**	39.6 33.5–45.5	44.3 35–52	0.105	42.8 35–51,5	45.5 37–54	**0.008**	45.5 36,5–54	45.5 37–54	0.987
**Gender**									
Male n, (%)	11 (91.6)	1214 (94.7)	0.642	112 (91.1)	1127 (94.9)	0.078	359 (93.4)	768 (95.5)	0.138
Female n, (%)	1 (8.3)	58 (4.5)	0.527	9 (7.3)	51 (4.3)	0.127	23 (6.0)	28 (3.5)	**0.046**
Other or unknown n, (%)	0 (0.0)	10 (0.9)	n.d	2 (1.6)	10 (0.8)	n.d	2 (0.5)	8 (1.0)	n.d
**Transmission group n, (%)**									
MSM n, (%)	11 (91.6)	1119 (87.3)	0.650	104 (84.6)	1045 (88.0)	0.274	329 (85.7)	716 (89.1)	0.094
PWID n, (%)	0 (0.0)	11 (0.9)	0.747	1 (0.8)	8 (0.7)	0.858	4 (1.0)	4 (0.5)	0.283
HET n, (%)	1 (8.3)	105 (8.7)	0.986	10 (8.1)	86 (7.2)	0.718	31 (8.1)	55 (6.8)	0.443
Other or unknown n, (%)	0 (0.0)	47 (3.7)	n.d	8 (6.5)	49 (4.1)	n.d	20 (5.2)	29 (3.6)	n.d

^a^Tested anti-N negative or anti-S1 negative

## Discussion

Serosurveys can provide valuable information on the spread of a virus and the vaccination coverage in a population of interest [33]. They are highly relevant during a pandemic for reaching decisions regarding essential public health measures and provide the data basis for mathematical modelling. Moreover, seroepidemiological studies facilitate retrospective analyses on the impact and success of implemented measures.

Since early in the COVID-19 surge it was assumed that the immune dysfunction caused by an HIV infection increases the vulnerability exerted by the coronavirus [17]. Public health authorities in Germany tried to mitigate the risk by prioritizing PLWH early in the vaccination campaign, but the outcome of this prioritization has not been investigated yet. Moreover, infection rates, seroprevalence data and vaccination coverage of PLWH in Germany were not analyzed in detail. With the HIVCOV study we therefore aimed to fill this gap.

During the pre-vaccination era in 2020 we found a fairly stable rate of 1.6% (anti-S1) and 1.2% (anti-N) of seropositive cases in the cohort that increased significantly in the last month of the era because of the second infection wave that started a few weeks earlier [30]. It is important to mention in this context that in our survey we are not gauging the cumulative incidence but the seroprevalence with a serologic test. Therefore, the antibody decay below the detection level of the ELISA-assays used has to be considered. The maintained seropositivity after a seroconversion is more robust for the anti-S1 response explaining the lower anti-N seroprevalence [34, 35]. Following the first infection wave in March and April 2020, the incidence rates in Germany were extremely low during the summer and early autumn months [31]. Our seroprevalence measured in the months before December 2020 are very similar to the data from the RKI-SOEP study, which investigated seroprevalence in the adult general population in Germany in October–November 2020 using the same anti-S1 ELISA. They report an anti-S1 seroprevalence of 1.3% [31]. Taking these results into account, our HIVCOV seroprevalence data do not indicate an increased risk of SARS-CoV-2 infection in this PLWH cohort at least during the initial phase of the pandemic. It has been reported that in some populations PLWH had a higher incidence of SARS-CoV-2 infections [25]. However, that discrepancy often vanished after adjusting for confounders such as socioeconomic and behavioral factors [19].

The effect of vaccination is clearly visible from February 2021 on. From that time on, the anti-S1 seroprevalence in our PLWH cohort is higher than the seroprevalences reported from nationwide and most regional or local serosurveys in adults (see Fig. 2). Our serological results are in line with information on early vaccinations of cohort members in January and February 2021 that we received from study centers. These finding indicate that PLWH in our cohort used the opportunity for prioritized vaccination, although the majority got vaccinated in April, May and June when already more vaccines were available. This resulted in a sharp increase of anti-S1 seroprevalence. The higher seroprevalence in comparison to nationwide serosurveys in adults shortly after the steep rise could also be a consequence of an earlier vaccination in PLWH. Later in 2022 the seroprevalence reached a plateau where only 2–3% of the samples were seronegative. A modelling study has estimated a proportion of the adult German population without infection and vaccination of 3.8% at the end of May 2022 [32]. Assuming a similar infection rate in PLWH and people living without HIV in the vaccination era, these results attest a similar SARS-CoV-2 vaccination coverage of PLWH in our cohort as in the general adult population. High acceptance rates of PLWH for vaccination were also reported from Spain (95.7%) [36] and the United Kingdom 96% [37]. High vaccination rates and booster vaccinations are important in PLWH especially for those with immune deficits as prior studies indicate that SARS-CoV-2 immunogenicity varies with the degree of immunosuppression [38, 39]. However, our data provide evidence for a lower vaccination rate in females compared to the group of all non-females during the study period. This observation might be associated with a higher proportion of migrants and other individuals with non-German origin among the females in the cohort. In migrants a lower vaccination rate has been reported in Germany in the COVIMO survey [40].

Because of the rapid waning of the anti-N response below the detection level of our ELISA [35] the proportion of anti-N positive cases does by no means represent the cumulative prevalence of past infections in the vaccination era. It is however useful for the analysis of infection frequencies in a population in the month before sampling. The rise and falls can be correlated with the incidence waves caused by emerging new variants monitored as notified infections. By doing this, we have observed that the anti-N-peaks lag about 2 months behind an incidence wave. As underlying reason, we assume that SARS-CoV-2 infected PLWH with a positive SARS-CoV-2 test result might have delayed or even skipped the routine quarterly visit in the HIV center. The next blood sample for routine checkups would have than be taken later.

The study is subject to certain limitations. First, the HIV-1 Seroconverter Cohort is not fully representative for PLWH in Germany. MSM, especially MSM from Berlin, are overrepresented and the number of women is particularly low and underrepresented demanding caution in interpretation of the results for this gender. Second, the number of samples analyzed per month varies significantly. Third, in the years 2021 and 2022 we analyzed a mixture of follow-up samples and samples from new cohort members. The time period between follow-ups was not always exactly one year.

## Conclusions

The average 1.6% anti-S1 seroprevalence in the cohort in 2020 was similar to data from the general population indicating a comparable incidence rate for PLWH and people without HIV. The increase in anti-S1 antibody seroprevalence in the first half of 2021 occurred somewhat earlier than in the general population. This increase was most likely caused by the high vaccination dynamic due to the prioritization of HIV positive individuals and by infections during the third wave. The anti-S1 seroprevalence in 2021 was slightly above the seroprevalence reported for the general population but the difference decreased subsequently. The results are in line with a high acceptance rate for SARS-CoV-2 vaccination in PLWH in Germany.

## Supplementary Information

Additional file 1. Details of specimen provision by participant. For each year of the study the number of participants contributing a sample to this and to the two other years is shown. 596 participants provided a sample in all three years of the study.

Additional file 2.

## Data Availability

Anonymized data and material are available upon reasonable request. Please send data access requests to the corresponding author.
