# Supplementary material for: SARS-CoV-2 seroprevalence among people living with HIV in the German HIV-1 Seroconverter Cohort, 2020–2022
Source: BMC Infect Dis. 2024 Nov 1;24:1228. doi: 10.1186/s12879-024-10119-3 (PMC11529177; doi:10.1186/s12879-024-10119-3)
Supplement: Supplementary file 1 — Additional file 1. Details of specimen provision by participant. For each year of the study the number of participants contributing a sample to this and to the two other years is shown. 596 participants provided a sample in all three years of the study. [file 12879_2024_10119_MOESM1_ESM.pdf]

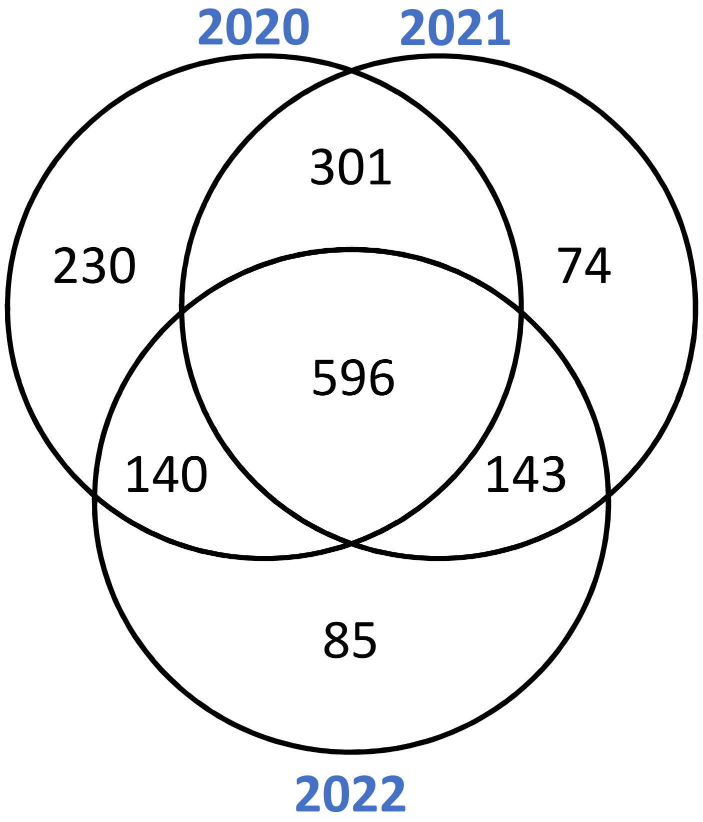

Additional file 1

Details of specimen provision by participant. For each year of the study the number of participants contributing a sample to this and to the two other years is shown. 596 participants provided a sample in all three years of the study.
