# Supplementary material for: SARS-CoV-2 seroprevalence among people living with HIV in the German HIV-1 Seroconverter Cohort, 2020–2022
Source: BMC Infect Dis. 2024 Nov 1;24:1228. doi: 10.1186/s12879-024-10119-3 (PMC11529177; doi:10.1186/s12879-024-10119-3)
Supplement: Supplementary file 2 — Additional file 2. [file 12879_2024_10119_MOESM2_ESM.pdf]

**Additional file 2 : Characteristics of monthly patient samples.**

| | Samples | Male | % | Female | % | Oth/Unkn Sex | % | MSM | % | PWID | % | HET | % | Oth/Unkn Tr | % |
|---|---|---|---|---|---|---|---|---|---|---|---|---|---|---|---|
| **Jan-20** | 126 | 120 | 95.2 | 5 | 4.0 | 1 | 0.8 | 113 | 89.7 | 0 | 0.0 | 8 | 6.3 | 5 | 4.0 |
| **Feb-20** | 106 | 102 | 96.2 | 4 | 3.8 | 0 | 0.0 | 90 | 84.9 | 0 | 0.0 | 9 | 8.5 | 7 | 6.6 |
| **Mar-20** | 93 | 90 | 96.8 | 3 | 3.2 | 0 | 0.0 | 85 | 91.4 | 3 | 3.2 | 3 | 3.2 | 2 | 2.2 |
| **Apr-20** | 97 | 93 | 95.9 | 4 | 4.1 | 0 | 0.0 | 89 | 91.8 | 0 | 0.0 | 4 | 4.1 | 4 | 4.1 |
| **May-20** | 81 | 76 | 93.8 | 4 | 4.9 | 1 | 1.2 | 71 | 87.7 | 0 | 0.0 | 8 | 9.9 | 2 | 2.5 |
| **Jun-20** | 118 | 112 | 94.9 | 5 | 4.2 | 1 | 0.8 | 105 | 89.0 | 2 | 1.7 | 7 | 5.9 | 4 | 3.4 |
| **Jul-20** | 190 | 181 | 95.3 | 9 | 4.7 | 0 | 0.0 | 158 | 83.2 | 3 | 1.6 | 19 | 10.0 | 10 | 5.3 |
| **Aug-20** | 126 | 116 | 92.1 | 9 | 7.1 | 1 | 0.8 | 107 | 84.9 | 1 | 0.8 | 15 | 11.9 | 3 | 2.4 |
| **Sep-20** | 93 | 83 | 89.2 | 7 | 7.5 | 3 | 3.2 | 78 | 83.9 | 1 | 1.1 | 11 | 11.8 | 3 | 3.2 |
| **Oct-20** | 106 | 101 | 95.3 | 4 | 3.8 | 1 | 0.9 | 97 | 91.5 | 0 | 0.0 | 8 | 7.5 | 1 | 0.9 |
| **Nov-20** | 73 | 71 | 97.3 | 2 | 2.7 | 0 | 0.0 | 63 | 86.3 | 1 | 1.4 | 6 | 8.2 | 3 | 4.1 |
| **Dec-20** | 58 | 55 | 94.8 | 2 | 3.4 | 1 | 1.7 | 50 | 86.2 | 0 | 0.0 | 6 | 10.3 | 2 | 3.4 |
| **Jan-21** | 103 | 96 | 93.2 | 5 | 4.9 | 2 | 1.9 | 91 | 88.3 | 1 | 1.0 | 7 | 6.8 | 4 | 3.9 |
| **Feb-21** | 95 | 94 | 98.9 | 1 | 1.1 | 0 | 0.0 | 83 | 87.4 | 0 | 0.0 | 7 | 7.4 | 5 | 5.3 |
| **Mar-21** | 85 | 79 | 92.9 | 5 | 5.9 | 1 | 1.2 | 74 | 87.1 | 1 | 1.2 | 3 | 3.5 | 7 | 8.2 |
| **Apr-21** | 96 | 91 | 94.8 | 4 | 4.2 | 1 | 1.0 | 81 | 84.4 | 0 | 0.0 | 10 | 10.4 | 5 | 5.2 |
| **May-21** | 94 | 87 | 92.6 | 7 | 7.4 | 0 | 0.0 | 80 | 85.1 | 1 | 1.1 | 8 | 8.5 | 5 | 5.3 |
| **Jun-21** | 97 | 95 | 97.9 | 2 | 2.1 | 0 | 0.0 | 91 | 93.8 | 0 | 0.0 | 4 | 4.1 | 2 | 2.1 |
| **Jul-21** | 169 | 155 | 91.7 | 12 | 7.1 | 2 | 1.2 | 141 | 83.4 | 3 | 1.8 | 16 | 9.5 | 9 | 5.3 |
| **Aug-21** | 105 | 97 | 92.4 | 8 | 7.6 | 0 | 0.0 | 87 | 82.9 | 1 | 1.0 | 12 | 11.4 | 5 | 4.8 |
| **Sep-21** | 74 | 71 | 95.9 | 3 | 4.1 | 0 | 0.0 | 67 | 90.5 | 0 | 0.0 | 6 | 8.1 | 1 | 1.4 |
| **Oct-21** | 93 | 88 | 94.6 | 4 | 4.3 | 1 | 1.1 | 84 | 90.3 | 0 | 0.0 | 8 | 8.6 | 1 | 1.1 |
| **Nov-21** | 69 | 66 | 95.7 | 2 | 2.9 | 1 | 1.4 | 64 | 92.8 | 0 | 0.0 | 3 | 4.3 | 2 | 2.9 |
| **Dec-21** | 34 | 32 | 94.1 | 2 | 5.9 | 0 | 0.0 | 30 | 88.2 | 0 | 0.0 | 2 | 5.9 | 2 | 5.9 |
| **Jan-22** | 98 | 92 | 93.9 | 3 | 3.1 | 3 | 3.1 | 91 | 92.9 | 0 | 0.0 | 5 | 5.1 | 2 | 2.0 |
| **Feb-22** | 84 | 82 | 97.6 | 2 | 2.4 | 0 | 0.0 | 74 | 88.1 | 2 | 2.4 | 4 | 4.8 | 4 | 4.8 |
| **Mar-22** | 66 | 64 | 97.0 | 2 | 3.0 | 0 | 0.0 | 62 | 93.9 | 0 | 0.0 | 2 | 3.0 | 2 | 3.0 |
| **Apr-22** | 66 | 60 | 90.9 | 6 | 9.1 | 0 | 0.0 | 57 | 86.4 | 0 | 0.0 | 7 | 10.6 | 2 | 3.0 |
| **May-22** | 79 | 76 | 96.2 | 3 | 3.8 | 0 | 0.0 | 71 | 89.9 | 1 | 1.3 | 2 | 2.5 | 5 | 6.3 |
| **Jun-22** | 84 | 78 | 92.9 | 5 | 6.0 | 1 | 1.2 | 73 | 86.9 | 0 | 0.0 | 6 | 7.1 | 5 | 6.0 |
| **Jul-22** | 110 | 105 | 95.5 | 5 | 4.5 | 0 | 0.0 | 99 | 90.0 | 1 | 0.9 | 6 | 5.5 | 4 | 3.6 |
| **Aug-22** | 121 | 113 | 93.4 | 7 | 5.8 | 1 | 0.8 | 95 | 78.5 | 1 | 0.8 | 16 | 13.2 | 9 | 7.4 |
| **Sep-22** | 82 | 79 | 96.3 | 2 | 2.4 | 1 | 1.2 | 75 | 91.5 | 0 | 0.0 | 4 | 4.9 | 3 | 3.7 |
| **Oct-22** | 74 | 71 | 95.9 | 3 | 4.1 | 0 | 0.0 | 66 | 89.2 | 1 | 1.4 | 3 | 4.1 | 4 | 5.4 |
| **Nov-22** | 64 | 61 | 95.3 | 3 | 4.7 | 0 | 0.0 | 56 | 87.5 | 0 | 0.0 | 6 | 9.4 | 2 | 3.1 |
| **Dec-22** | 36 | 35 | 97.2 | 1 | 2.8 | 0 | 0.0 | 31 | 86.1 | 1 | 2.8 | 3 | 8.3 | 1 | 2.8 |
| **2020-2022** | **3345** | **3167** | **94.7** | **155** | **4.6** | **23** | **0.7** | **2929** | **87.6** | **25** | **7.5** | **254** | **0.7** | **137** | **4.1** |

Abbreviations: Oth, Other; Unkn, Unknown; Tr, Transmission; MSM, men who have sex with men; PWID, persons who inject drugs; HET, heterosexual transmission.
